# Prediction of Severe Neonatal Hyperbilirubinemia Using Cord Blood Hydrogen Peroxide: A Prospective Study

**DOI:** 10.1371/journal.pone.0086797

**Published:** 2014-01-23

**Authors:** Hung-Chieh Chou, Chiang-Ting Chien, Po-Nien Tsao, Wu-Shiun Hsieh, Chien-Yi Chen, Mei-Hwei Chang

**Affiliations:** 1 National Taiwan University College of Medicine Graduate Institute of Clinical Medicine, Taipei, Taiwan; 2 Department of Pediatrics, National Taiwan University Hospital, National Taiwan University, Medical College, Taipei, Taiwan; 3 Department of Life Science, National Taiwan Normal University, Taipei, Taiwan; Catholic University Medical School, Italy

## Abstract

**Background:**

We hypothesized that cord blood hydrogen peroxide (H_2_O_2_) could be utilized to predict the severity of neonatal hyperbilirubinemia.

**Methods:**

We prospectively enrolled term or near-term healthy neonates. Cord blood and capillary blood at three days of age were measured for hydrogen peroxide and bilirubin concentrations. For newborns with hyperbilirubinemia, further blood samples were obtained at five and seven days of age. Newborns were divided into severe or less severe hyperbilirubinemic groups (peak bilirubin ≥17 mg/dL or not). The sensitivity, specificity, and negative predictive values were determined.

**Results:**

There were 158 neonates enrolled. The incidence of neonatal hyperbilirubinemia was 30.5% for a concentration ≥15 mg/dl. The rising patterns were similar among bilirubin concentrations and hydrogen peroxide levels during the first few days of life. There was a strong positive correlation between bilirubin concentrations and hydrogen peroxide levels after correlation analysis. The rate of severe hyperbilirubinemia was 13.3%. It revealed that a cord blood hydrogen peroxide signal level of 2500 counts/10 seconds was an appropriate cut-off for predicting severe hyperbilirubinemia. Sensitivity and the negative predictive value were 76.2% and 93.3%, respectively.

**Conclusions:**

Our findings confirm that hydrogen peroxide levels and bilirubin concentrations in cord and neonatal blood are closely related. A cord blood hydrogen peroxide level above 2500 counts/10 seconds associated with a high predictive value for severe hyperbilirubinemia. This method provides information about which neonate should be closely followed after discharge from the nursery.

## Introduction

The incidence of neonatal jaundice is around 60∼70% in Western countries [1]. and even higher among newborns of Asian ethnicity. Neonatal jaundice is associated with increased unconjugated bilirubin concentrations caused by the breakdown of red blood cells. Bilirubin can damage neurologic tissue and lead to bilirubin-induced neurologic dysfunction [2]–[4]. Bilirubin in itself is not completely detrimental and can exert a physiological protective effect [5] due to its antioxidant properties [6]. A number of previous studies have highlighted the relationship between bilirubin and nitric oxide, reactive oxygen/nitrogen species [7]–[11].

The American Academy of Pediatrics published guidelines for the management of neonatal jaundice in 1994 [12]. Since then, several cases of kernicterus have been reported [13]. Another prominent concern is that neonates may be discharged from hospital too early, before the onset of neonatal jaundice. This may delay the diagnosis of severe neonatal hyperbilirubinemia and thereby increase the incidence of kernicterus [14]. Therefore, researchers have been enthusiastic about identifying predictors of neonatal hyperbilirubinemia to assist in the early detection of neonates at high risk of severe hyperbilirubinemia. The hour-specific bilirubin nomogram [15] is widely accepted by most clinicians, but has a low sensitivity and may vary by ethnicity.

Given the relationship between bilirubin and hydrogen peroxide, which is one of reactive oxygen species, we hypothesized that hydrogen peroxide levels in cord blood could be used to predict neonatal hyperbilirubinemia.

## Methods

### Subjects

All term or near-term healthy neonates (gestational age ≥34 weeks) born at National Taiwan University Hospital during May and June of 2005 were candidates for enrollment into this prospective follow-up study. InfantsNewborns with early onset hyperbilirubinemia (bilirubin ≥10 mg/dL before 24 hours of age), maternal ABO incompatibility, glucose-6-phosphate-dehydrogenase deficiency, cephalohematoma, multiple congenital anomalies, maternal gestational diabetes mellitus, anemia, congenital hypothyroidism, sepsis, cholestasis, and urinary tract infection were excluded. All procedures were approved and supervised by the Ethics Committee of Clinical Trials of National Taiwan University Hospital (9361700383). Signed informed consent forms were collected from parents before enrollment.

Peak bilirubin concentrations were determined in all infantsnewborns during the follow-up period. All infantsnewborns with bilirubin concentrations ≥15 mg/dL were admitted for phototherapy; bilirubin concentrations were monitored until they fell below 13 mg/dL.

### Sample Collection

A total of 10 mL of cord blood was obtained from all enrolled infantsnewborns at birth. Capillary blood was also collected in a microtube using the heel-stick lancet method on the third day after birth. For infantsnewborns admitted with hyperbilirubinemia, capillary blood was obtained at five and seven day of age. All samples were processed immediately.

### Bilirubin Measurement

All blood samples, including cord blood, were centrifuged at 1500 rpm for 5 minutes. Plasma bilirubin concentrations were then measured using a bilirubin meter (BR-501, APEL, Japan). If the bilirubin concentration was found to be >15 mg/dL, the sample was reanalyzed using a biochemical method in our central laboratory. Severe neonatal hyperbilirubinemia was indicated by a bilirubin concentration ≥17 mg/dL. At our institution, these infantsnewborns receive intensive phototherapy.

### Measurement of Specific Blood Hydrogen Peroxide Activity

Cord and capillary blood hematocrit levels were determined after centrifugation at 1500 rpm for 5 minutes. Chemiluminescence (CL) signal emitting H2O2 and CL-emitting substances (luminol, Sigma, St. Louis, MO) from whole blood (with phosphate buffered as a background control) were measured by use of a Chemiluminescence Analyzing System (CLD-110, Tohoku Electronic Industrial Co., Sendai, Japan). The blood samples were immediately wrapped in aluminum foil and kept on ice until CL measurement, usually done within 2 h. Immediately before CL measurement, 0.1 ml of phosphate-buffered saline (pH 7.4) was added to 0.1 ml of blood sample as described previously [16]. The CL was measured in a completely dark chamber of the Chemiluminescence Analyzing System. After 100-s background level determination, 1.0 ml of 0.1 mM luminol in phosphate-buffered saline (pH 7.4) was injected into the sample. The CL was monitored continuously for an additional 600 s. The total amount of CL was calculated by integrating of the area under the curve and subtracting it from the background level. The assay was performed in duplicate for each sample and was expressed as CL (H2O2) counts/10 s for blood CL. The mean ± SEM of the CL level of each sample was calculated.

### Statistical Analysis

The relationship between bilirubin and H2O2 signal from infant (up to five days of age) cord blood samples was determined by assessing the Pearson Correlation. Correlation analysis was performed and tested by regression analysis in each sample. We divided the eligible population into the groups by the bilirubin concentration (bilirubin ≥17 mg/dL or not). All parametric data were compared by independent samples t-test, whereas categorical data were compared by chi-square test. A receiver operating characteristic (ROC) curve was generated to determine a cut-off value for predicting severe neonatal hyperbilirubinemia. Sensitivity, specificity, and negative predictive values were calculated. A P value <0.05 was considered to indicate statistical significance.

## Results

During the study period, 282 healthy newborns were born at our institution. Of these newborns, 158 were enrolled in our study. The incidence of neonatal hyperbilirubinemia during follow-up was 48.7% for peak bilirubin ≥13 mg/dL and 30.5% for peak bilirubin ≥15 mg/dL. The incidence of severe hyperbilirubinemia (bilirubin ≥17 mg/dL) was 13.3%.

### The Relationship between Bilirubin Concentration and H_2_O_2_ Levels

**Figure 1:**
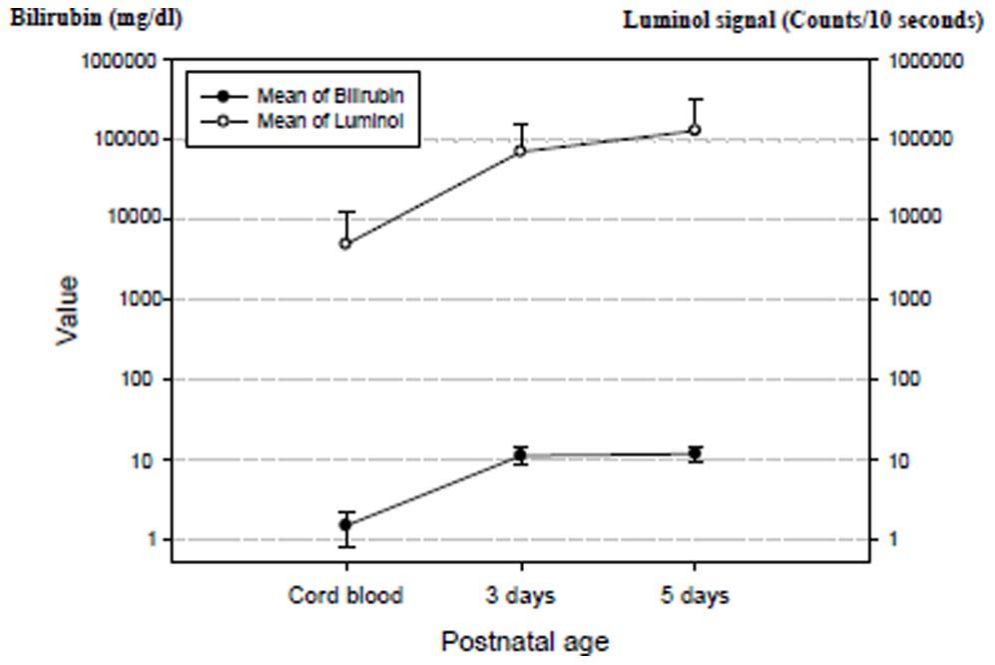
Bilirubin concentrations and hydrogen peroxide levels increased similarly during the first few days. The correlation between these two variables was 0.454, *P*<0.001. The left y-axis is the bilirubin concentration, whereas the right y-axis is the H_2_O_2_ (luminol) signal. The y-axis scale is logarithmic because of the high luminal signal values.

Bilirubin concentrations were significantly increased in three day-old newborns. The chemical illuminate H_2_O_2_ count was significantly increased in three and five day-old newborns (both *P*<0.0001). As postnatal age increased, there was a similar pattern of increase in bilirubin concentrations and H_2_O_2_ (Figure 1). There was a significant similar rising pattern (0.454, *P*<0.0001) between bilirubin and H_2_O_2_ levels.

**Figure 2:**
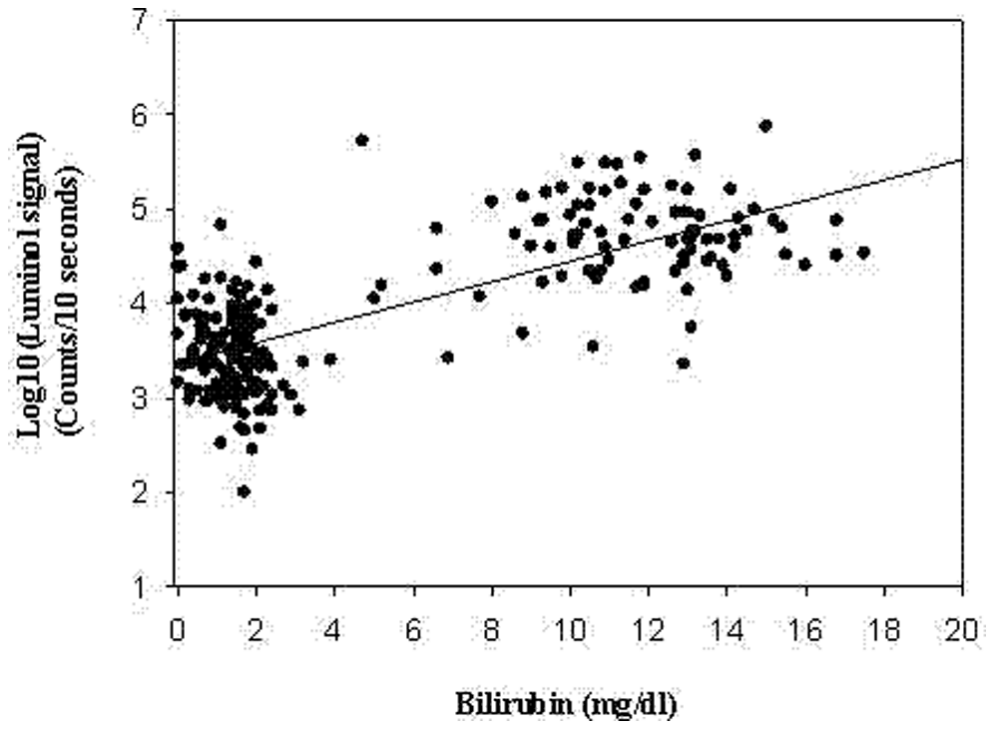
A scatter plot figure with bilirubin (on the X-axis) and the corresponding hydrogen peroxide levels with Logarithmic transformation (on the Y-axis) was presented. There is a linear relationship between these 2 values. The regression equation is H_2_O_2_ signal = 3.371+0.108*Bilirubin. The P value is <0.0001.

After the correlation analysis with scatter plot for each individual sample, there was a strong positive correlation between bilirubin concentrations and hydrogen peroxide levels (Figure 2). The p value was <0.0001.

### The Most Suitable Cut-off Value for Predicting Severe Neonatal Hyperbilirubinemia

**Figure 3:**
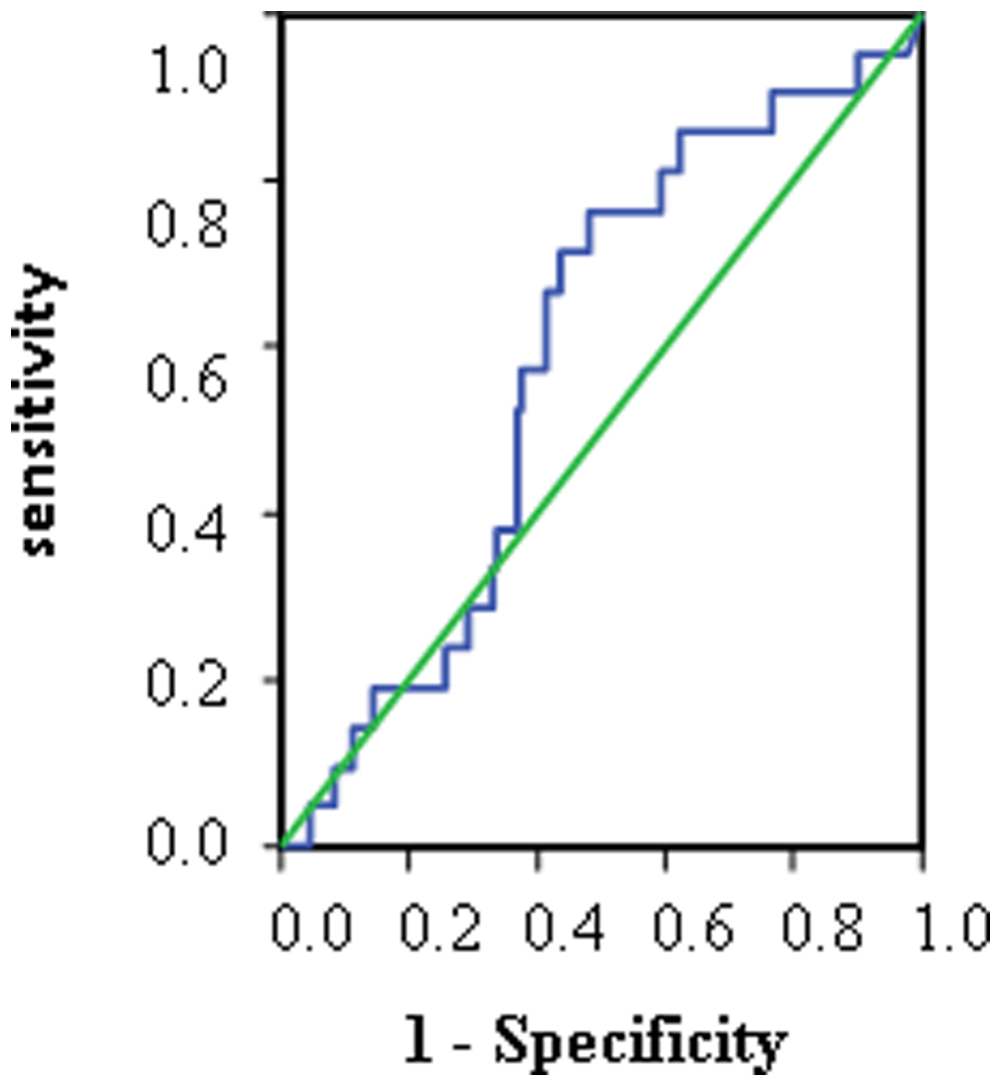
Receiver operating characteristic (ROC) curve of cord blood hydrogen peroxide (H_2_O_2_) signals at the peak bilirubin concentration (≥17 mg/dL).

We compared demographic and clinical characteristics between the severe hyperbilirubinemic group and the less severe hyperbilirubinemic group. There were no between group differences in gestational age, birth body weight, Apgar score (first and fifth minutes), cord blood bilirubin concentration, or cord blood hematocrit (Table 1). A comparison of the ROC curves for cord blood H_2_O_2_ signal levels between the severe and less severe hyperbilirubinemic groups (Figure 3) revealed that a cord blood H_2_O_2_ signal level of 2500 counts/10 seconds was the most suitable cut-off value for predicting severe neonatal hyperbilirubinemia. This cut-off was associated with 76.2% sensitivity and 54.8% specificity. We used three different cut-off H_2_O_2_ signal levels (2400, 2500, 2650 counts/10 seconds) to compare the severe and less severe hyperbilirubinemic groups (Table 2). All three cut-off values were found to be significant predictors of severe hyperbilirubinemia (*P* = 0.027, 0.017, and 0.030, respectively).

**Table 1:** Demographic and cord blood data for the severe and less severe hyperbilirubinemic groups.

	Less Severe hyperbilirubinemic Group (n = 133)^a^	Severe hyperbilirubinemic Group (n = 25)^b^	*P* Value
GA (weeks)	38.5±1.6	38.8±1.5	0.503
BBW (g)	3106.5±536.9	3246.6±395.1	0.302
Apgar score (1)	9 (4–9)	9 (6–9)	0.209
Apgar score (5)	9 (8–9)	9 (9–9)	0.488
Cord blood bilirubin (mg/dL)	1.40±0.67	1.52±0.80	0.740
Cord hematocrit (%)	43.10±5.77	44.63±7.14	0.165

Abbreviations: GA, gestational age; BBW, birth body weight.

a Newborns with peak bilirubin concentration <17 mg/dL during the postnatal follow-up period.

b Newborns with peak bilirubin concentration ≥17 mg/dL during the postnatal follow-up period.

**Table 2:** The different cut-off values of H_2_O_2_ signal for predicting severe hyperbilirubinemia were compared between the severe hyperbilirubinemic group and the less severe hyperbilirubinemic group.

Cord Blood Luminol Signal (Counts/10 seconds)	Sensitivity	Specificity	*P* Value*
2000	76.2%	40.6%	0.567
2200	76.2%	43.6%	0.443
2400	76.2%	49.6%	0.027
2500	76.2%	51.9%	0.017
2600	71.4%	53.4%	0.030
2800	71.4%	56.4%	0.157
2900	66.7%	56.4%	0.307

* Comparison was performed by chi-square test.

There were 75 newborns who had cord blood H_2_O_2_ signal levels that were below 2500 counts/10 seconds. Of these newborns, only five newborns developed severe hyperbilirubinemia during the follow-up period. Hence, the negative predictive value was a maximum of 93.3%. To define if any other risk factors of the 5 newborns who developed severe hyperbilirubinemia, despite having low hydrogen peroxide levels, we compared the demographic and clinical characteristics of these five newborns and with those of the less severe hyperbilirubinemic group (Table 3). Three newborns (cases 1, 3, and 5) had high initial bilirubin concentrations in cord blood. Three newborns (cases 2, 3, and 4) had high initial hematocrit values. One infant (case 5) exhibited the most pronounced loss in body weight during the first few days of life. All these five newborns possessed at least one factor that was beyond the range of one standard deviation from the mean value.

**Table 3:** Comparison of demographic and cord blood data between newborns in the less severe hyperbilirubinemic group and the five newborns who developed severe hyperbilirubinemia despite having a low cord blood hydorgen peroxide level.

Case no.	GA (weeks)	BW loss (%)	Cord Blood Bilirubin (mg/dL)	Cord Blood Hematocrit (%)
1	40	7.0	1.8↑*	43
2	39	6.0	1.5	52↑
3	39	9.1	2.0↑	49↑
4	40	7.8	1.5	50↑
5	38	11.1↑	1.8↑*	42
Less severe icteric group (n = 133)	38.5±1.6	7.8±2.7	1.40±0.67	43.1±5.77

Abbreviations: GA, gestational age; BW, body weight.

BW loss: physiological body weight loss = (birth weight−lowest body weight)/birth weight.

↓ or ↑: beyond mean ±1 standard deviation when compared with the less severe icteric group.

↑*: bilirubin concentration beyond mean ±0.5 standard deviation when compared with the less severe hyperbilirubinemic group.

## Discussion

We have found that there is a strong correlation between cord blood hydrogen peroxide(H_2_O_2_) levels and bilirubin concentrations in the immediate neonatal period. This is the first clinical study to examine the direct association between hydrogen peroxide levels and bilirubin concentrations. Our results suggest that cord blood hydrogen peroxide levels can be used to predict neonatal severe hyperbilirubinemia. Being able to identify newborns at birth who are likely to develop subsequent severe hyperbilirubinemia would be of obvious clinical benefit.

The greatest change after birth is the rapid transition from a low oxygen environment (fetus) to a high oxygen environment (neonate). During this change in environment, the oxygen concentration increases more than three-fold. Oxidation plays a role in the delivery process [17], [18]. Antioxidants levels remain low at birth; however, this induces a series of RBC breakdown and heme metabolic processes. There is an association between bilirubin concentrations and reductive and oxidative reactions.

The CL signals emitted by whole blood and CL-emitting substance (luminol), have been shown to be useful for detecting H_2_O_2_ [16]. A higher H_2_O_2_ signal indicates lower anti-oxidant activity and/or a decreased capacity for reactive oxygen species scavenging. We found that the level of hydrogen peroxide increases rapidly during the first few days of life, parallel with the increase in bilirubin concentrations. The significant correlation between hydrogen peroxide levels and bilirubin concentration supports our hypothesis. The increase in hydrogen peroxide levels is caused by increasing oxygen levels in the transition from fetal to the extra-fetal environment. Hydrogen peroxide may damage red blood cell membranes, leading to the production of bilirubin [19]. The greater the reactive oxidative product, such as hydrogen peroxide level after birth, the more bilirubin is produced. Hence, we can measure hydrogen peroxide levels as an indicator of severe hyperbilirubinemia.

As previously mentioned, bilirubin is an antioxidant of reactive oxygen/nitrogen species or nitric oxide [18], [20]. Bilirubin can be re-used as a substrate. Ideally, there will be a balance between oxidant and antioxidants levels. Therefore, if the reactive oxygen species levels increase beyond the antioxidant capability of bilirubin, bilirubin concentrations will increase further. This suggests that the relationship between hydrogen peroxide levels and bilirubin concentrations is not a direct proportion. That is why we cannot use it to predict less severely hyperbilirubinemic neonate.

As early nursery discharge is encouraged to facilitate bonding between the mother and newborn infant, early prediction of severe hyperbilirubinemia is of vital importance. The widely used hour-specific nomogram, described by Bhutani et al., only 68 among 172 newborns in high risk zone remained in the zone later, which means only 39.5% of sensitivity for predicting severe hyperbilirubinemia [15]. Low sensitivity is a major challenge in clinical research of prediction of neonatal hyperbilirubinemia. In the present study, the described means of predicting severe hyperbilirubinemia was associated with 76.2% sensitivity, far higher than that associated with the conventional nomogram. Further, we found that a cord blood H_2_O_2_ signal level >2500 counts/10 seconds was a good predictor of severe hyperbilirubinemia. This cut-off has a high negative predictive value (93.3%), which suggests that if the cord blood hydrogen peroxide level is <2500 counts/10 seconds, the chance of developing a dangerous bilirubin concentration (≥17 mg/dL) in the following days will be very low.

There were five newborns that developed severe hyperbilirubinemia despite having low cord hydrogen peroxide levels. Therefore, there are clearly other factors involved in the development of severe hyperbilirubinemia; hence, there is currently no single factor with a high sensitivity for predicting severe hyperbilirubinemia. It may be necessary to incorporate other risk factors, such as cord blood bilirubin concentrations and hematocrit levels or body weight loss in early life, to increase the sensitivity for detecting hyperbilirubinemia [21].

In conclusion, we have found that there is a strong correlation between cord blood hydrogen peroxide levels and bilirubin concentrations. The rising patterns were similar among bilirubin concentrations and hydrogen peroxide levels in the immediate neonatal period, both of which increase in concert during the first several days of life. A cord blood H_2_O_2_ signal level cut-off of 2500 counts/10 seconds can be used as a good predictor of severe hyperbilirubinemia with a high negative predictive value. This method provides which neonate should be followed after discharge from the nursery.
